# Non-invasive visualisation and identification of fluorescent *Leishmania tarentolae* in infected sand flies

**DOI:** 10.12688/wellcomeopenres.14910.1

**Published:** 2018-12-13

**Authors:** Hector M. Diaz-Albiter, Clément Regnault, Edubiel A. Alpizar-Sosa, Dagmara McGuinness, Michael Barrett, Rod J. Dillon

**Affiliations:** 1 El Colegio de la Frontera Sur, Villahermosa, Tabasco, 86280, Mexico; 2 Wellcome Centre for Molecular Parasitology, University of Glasgow, Glasgow, G12 8TA, UK; 3 Faculty of Health and Medicine, Lancaster University, Lancaster, Lancashire, LA1 4YQ, UK

**Keywords:** Leishmania, Lutzomyia, sand fly, fluorescence, GFP, parasite-vector interactions

## Abstract

**Background:** The leishmaniases are neglected diseases that affect some of the most vulnerable populations in the tropical and sub-tropical world. The parasites are transmitted by sand flies and novel strategies to control this neglected vector-borne disease are needed. Blocking transmission by targeting the parasite inside the phlebotomine vector offers potential in this regard. Some experimental approaches can be best performed by longitudinal study of parasites within flies, for which non-destructive methods to identify infected flies and to follow parasite population changes are required.

**Methods:** *Lutzomyia longipalpis* were reared under standard insectary conditions at the Wellcome Centre for Molecular Parasitology. Flies were artificially infected with *L. tarentolae* expressing green fluorescent protein (GFP. Parasite counts were carried out 5 days post-infection and the percentage of infected flies and survival of infected females was established up to days 5 post-infection. Whole living females were visualised using an epifluorescence inverted microscope to detect the presence parasites inferred by a localised green fluorescent region in the upper thorax. Confirmation of infection was performed by localised-fluorescence of dissected flies and estimates of the parasite population.

**Results** **:** *Leishmania tarentolae* was successfully transfected and expressed GFP *in vitro*. *L. tarentolae*-GFP Infected flies showed similar parasite populations when compared to non-transfected parasites ( *L. tarentolae*-WT). Survival of non-infected females was higher than *L. tarentolae*-infected groups, (Log-rank (Mantel-Cox) test, p<0.05). *L. tarentolae*-GFP infected females displayed an intense localised fluorescence in the thorax while other specimens from the same infected group did not. Localised fluorescent flies were dissected and showed higher parasite populations compared to those that did not demonstrate high concentrations in this region (t-test, p<0.005).

**Conclusion** **:** These results demonstrate the feasibility of establishing a safe non-human infectious fluorescent *Leishmania*-sand fly infection model by allowing non-destructive imaging to signal the establishment of Leishmania infections in living sand flies.

## Introduction

Leishmania are protozoan parasites belonging to the Order Kinetoplastida. They infect various vertebrate species and are transmitted via the bite of phlebotomine sand flies ( Bates, 2007). Approximately 20 species of this genus cause the leishmaniases in humans, a group of diseases presenting different clinical manifestations, some of which can lead to death ( Burza *et al.*, 2018). The disease is endemic in 97 countries reporting approximately 1 million new cases yearly ( WHO, 2018). Although, some treatments are available, they are associated with high toxicity and quite often administered too late and treatment failures are common. No vaccine is available and overuse of pesticides to control arthropod populations worldwide has resulted in development of resistance in arthropod vectors, including sandflies that transmit *Leishmania* ( Hemingway & Ranson, 2000; Hassan *et al.*, 2012).

Alternative approaches to intervening against the leishmaniases are sought and novel approaches to transmission control are attractive ( Beaty, 2000). Novel strategies aiming to develop new approaches to interrupt the transmission cycle rely on a thorough knowledge of insect-parasite interactions ( Kamhawi, 2006). Several studies focusing on *Leishmania* metabolism, drug resistance, and host-parasite interactions led to us establishing a colony of *Lutzomyia longipalpis* sandflies at the Wellcome Centre for Molecular Parasitology (WCMP). *Lutzomyia longipalpis* is permissive to infection with multiple *Leishmania* species ( Soares & Turco, 2003; Volf & Myskova, 2007), making it a versatile model for laboratory based work on parasite-vector interactions.

The *Leishmania*-sand fly model established at the WCMP was tested for potential in establishing artificial infections using the non-human pathogenic *Leishmania tarentolae*. This species was first identified in the gecko *Tarentola mauritanica* ( Adler & Theodor, 1935; Chatton & Blanc, 1914; Laveran & Franchini, 1921). Both promastigote and (rarely) amastigote forms are found circulating in the blood, whereas promastigotes are located in the intestinal lumen as well as the cloacae of their lizard hosts ( Wilson & Southgate, 1979). The natural vectors of this species are *Sergentomyia* spp., and its development in this fly has been described as hypopylarian, i.e. a parasite that establishes in the hindgut of the sand fly ( Bates, 2007). Also, DNA of *L. tarentolae* has been also found in *Phlebotomus argentypes* ( Gajapathy *et al.*, 2013). However, very little is known about its biology inside the vector. Research on this species has focussed mainly on its use as a model to study antibiotic resistance and metabolism ( Aphasizhev *et al.*, 2003; Dey *et al.*, 1994; Ouellette *et al.*, 1990), and more recently, it has become a well-establish, commercially available expression system ( Breitling *et al.*, 2002; Kushnir *et al.*, 2005; Zhang *et al.* 1995).

We transfected the parasite to express GFP aiming to perform further experiments visualising parasite dynamics in the sand fly gut, infections with antibiotic-resistant parasites, as well as to provide a safe protocol for training students and staff to perform *Leishmania* infections where containment facilities are not required.

We showed that non-transfected and GFP-expressing *L. tarentolae* were present in the gut 5 days post infection. It was observed that GFP-parasite infected flies presented a strong fluorescence area in the thorax under the microscope before dissecting them, which was the midgut. We confirmed that flies presenting this localised fluorescence were heavily infected. The technique presented here provides, for the first time, the ability to study the Leishmania-sandfly relationship in a non-destructive fashion. This non-destructive GFP infection signalling can be used for testing chemicals that might kill parasites within the vector, infected fly behaviour experiments determining microbiome-relationships that might prevent transmission.

## Methods

### Sand fly rearing

A colony of *Lutzomyia longipalpis* was initiated from flies originating from the colony at Lancaster University and further flies provided from the lab of Paul Kaye (University of York), both derived originally from Jacobina, Brazil. Insects were kept under standard laboratory conditions ( Modi & Tesh, 1983). Briefly, all developmental stages were kept at 27°C (±2°C), a relative humidity of ~80% and a photoperiod of 8 hours light/16 hours darkness. Larvae were kept in plastic pots with a plaster substrate and fed a 1:1 rabbit food and droppings mixture that was autoclaved and ground. Adults were fed *ad libitum* with 70% w/v sucrose solution offered in cotton wool. Females were fed on commercially obtained sheep blood (in Alsever’s as anticoagulant) via a Hemotek membrane feeder (Discovery Workshops, UK) at 37°C.

### Parasite cultures

*Leishmania tarentolae* (strain p10) promastigotes were kindly donated by Barrie Rooney (University of Kent) and cultured in HOMEM medium (GE Healthcare) supplemented with 10% heat-inactivated foetal bovine serum (HiFBS, Gibco) and 1% penicillin/streptomycin solution (Sigma-Aldrich). Cells were maintained by weekly sub-passage of stationary-phase cultures in fresh complete HOMEM at a density of 10 ^5^–10 ^6^ cells/ml. Cultures were kept in non-vented T25 or T75 flasks (Corning) and incubated at 25°C.

### Transfections

Transfections were performed on mid-log phase *L. tarentolae* promastigotes. A total of 5 × 10 ^7^ parasites were pelleted (1200xg, 10 min) and washed once with ice-cold PBS before resuspending in 100 μl of transfection buffer (90 mM NaPO _4_, 5 mM KCl, 50 mM HEPES, 0.15 mM CaCl _2_, pH7.3). The suspension was transferred to a 1 ml Bio-Rad GenePulser® cuvette, and mixed with 10 μg of plasmid pGL1132 DNA in 20 μl sterile water, or 20 μl of sterile water as a negative control. Parasites were electroporated using an Amaxa NucleofectorTM II and subsequently transferred to 10 ml of complete HOMEM in a T25 non-vented flask (Corning). After an overnight incubation at 25°C, appropriate drugs (G418 or Hygromycin, 25 μg/ml) were added in the culture to select for transfectants. Successful transfection was evaluated by confirmation of fluorescence using a Carl Zeiss Axiovert 40 CFL inverted epifluorescence microscope (488 nm excitation and 509 nm emission).

### Sand fly infections

A total of 2 × 10 ^6^ parasites were pelleted (1200xg, 10 min) and washed once with ice-cold PBS before resuspending in 1ml of blood meal (sheep blood mixed with heat-inactivated serum) and fed to the insects using a chick skin membrane with the Hemotek apparatus at 37°C as described above. Groups of ~80 females were fed for one hour, after which 60 fully-engorged individuals were separated and kept under standard laboratory conditions until required for experimental work. Negative control groups were flies fed only on sheep blood and manipulated as stated above. Sandfly mortality was recorded daily and 14 insects were dissected 5 days post infection (DPI), when defecation had already taken place and parasites have likely established an infection. Parasite counts were performed by dissecting individual midguts in PBS and manually homogenizing them in 50 ul of PBS. Total parasites were counted under the microscope using a haemocytometer. Percentage of infected flies 5 DPI was recorded and sandfly survival was evaluated every day for the duration of the experiment. Three biological replicates were performed.

### Assessment of fluorescence *in vivo*

An intense green fluorescence in the thorax area *of L. tarentolae*-GFP infected flies was observed, 14 individuals were then sorted out into 2 groups based on the absence and presence of this localised fluorescence. Midguts were dissected as described above, confirmation of fluorescent parasites inside intact guts was performed using a Carl Zeiss Axiovert 40 CFL inverted epifluorescence microscope (488 nm excitation and 509 nm emission) and parasite counts of each gut were performed using a haemocytometer as described above.

### Statistical analysis

Survival analyses were performed using the Kaplan-Meier Log Rank χ2 test. Groups were compared using unpaired t-test and infection percentages were compared using a Fisher’s test. Results are expressed as the group mean ± SEM. Significance was considered when p < 0.05. All data were analysed with GraphPad Prism (version 5.00, Prism Inc).

## Results

### Sandfly infections with GFP-transfected *L. tarentolae*

**Figure 1.**
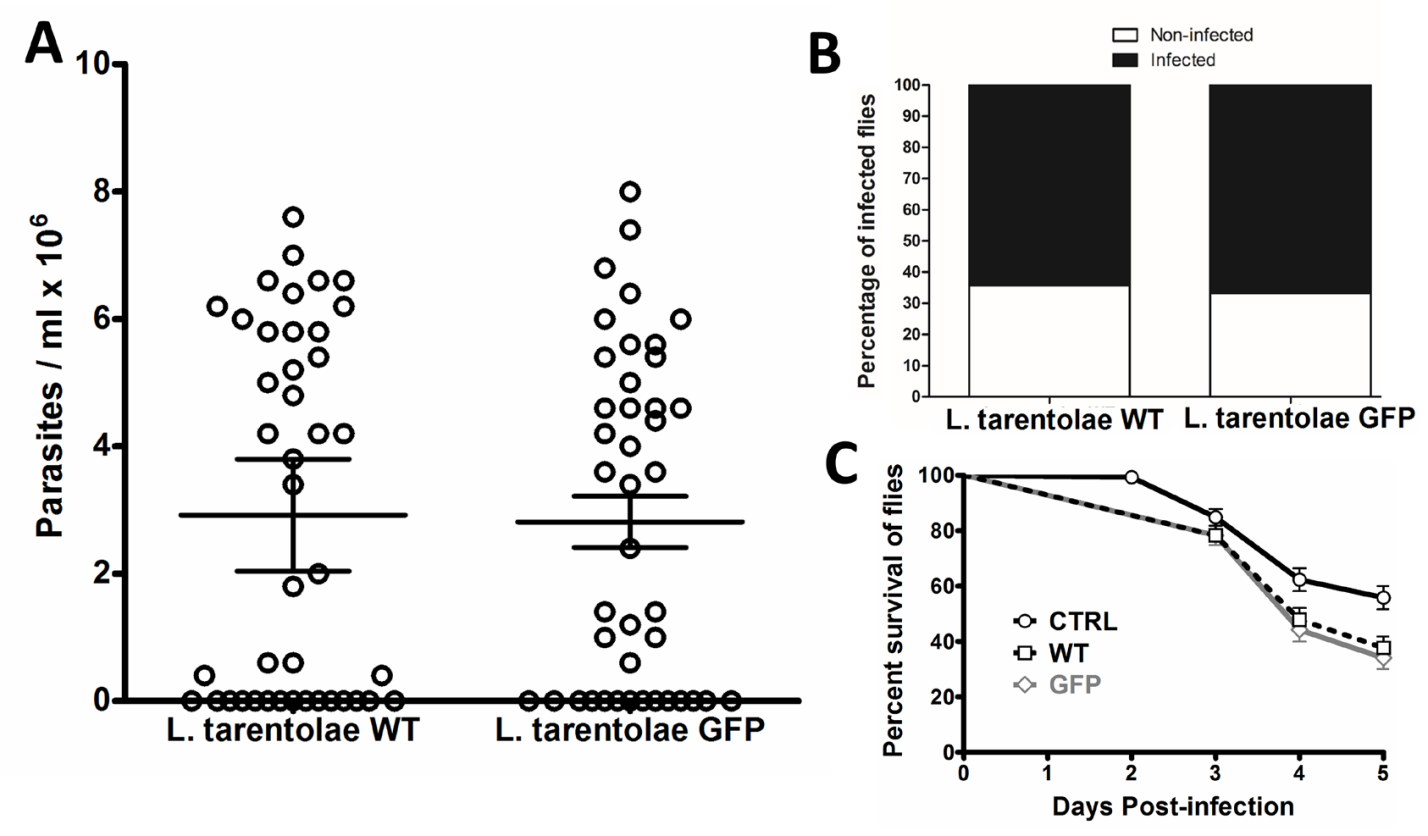
Artificial Infection of female *Lutzomyia longipalpis* with *Leishmania tarentolae*. ( **A**) Scatter plot shows estimates of parasite populations of non-transfected parasites *(L. tarentolae*-WT) and GFP transfected parasites ( *L. tarentolae*-GFP) infected flies 5 days post infection (DPI). Each circle represent parasite counts in individual flies, bars are mean ± SEM. ( **B**) Bars represent percentage of infected (black) and non-infected (white) flies 5 DPI. ( **C**) Survival curves of *L. tarentolae*-WT, *L. tarentolae*-GFP and non-infected negative control. Survival curves of WT and GFP were not statistically different, negative control showed significant differences compared with WT and GFP (Log-rank (Mantel-Cox) Test, p<0.05).

Transfection of *L. tarentolae* with plasmid pGL1132 and subsequent expression of GFP was successfully achieved *in vitro* in *L. tarentolae* ( Supplementary Figure 1). Fluorescent parasites were tested alongside WT parasites to determine infectivity in flies. Estimates of parasite population from dissected female *L. longipalpis* infected with *L. tarentolae*-GFP showed no significant differences (unpaired t-test, p=0.8602) compared with non-transfected parasites ( *L. tarentolae*-WT) 5 days post-infection ( Figure 1A). Percentage of *L. tarentolae*-GFP vs. *L. tarentolae*-WT dissected on day 5 post-infection were not statistically different (F test, p=0.6298; Figure 1B). Sand fly mortality was evaluated over the length of the experiments and data are presented as survival curves from *L. tarentolae*-GFP, *L. tarentolae*-WT and blood-fed, non-infected negative control insects ( Figure 1C). Survival curves were not statistically different between infected groups (Log-rank (Mantel-Cox) Test, p=0.5801). However, survival of non-infected, negative control group was higher and significantly different compared against *L. tarentolae*-GFP and *L. tarentolae*-WT infected flies (Log-rank (Mantel-Cox) Test, p<0.005, p<0.05, respectively).

### Assessment of *L. tarentolae*-GFP fluorescence *in vivo*

**Figure 2.**
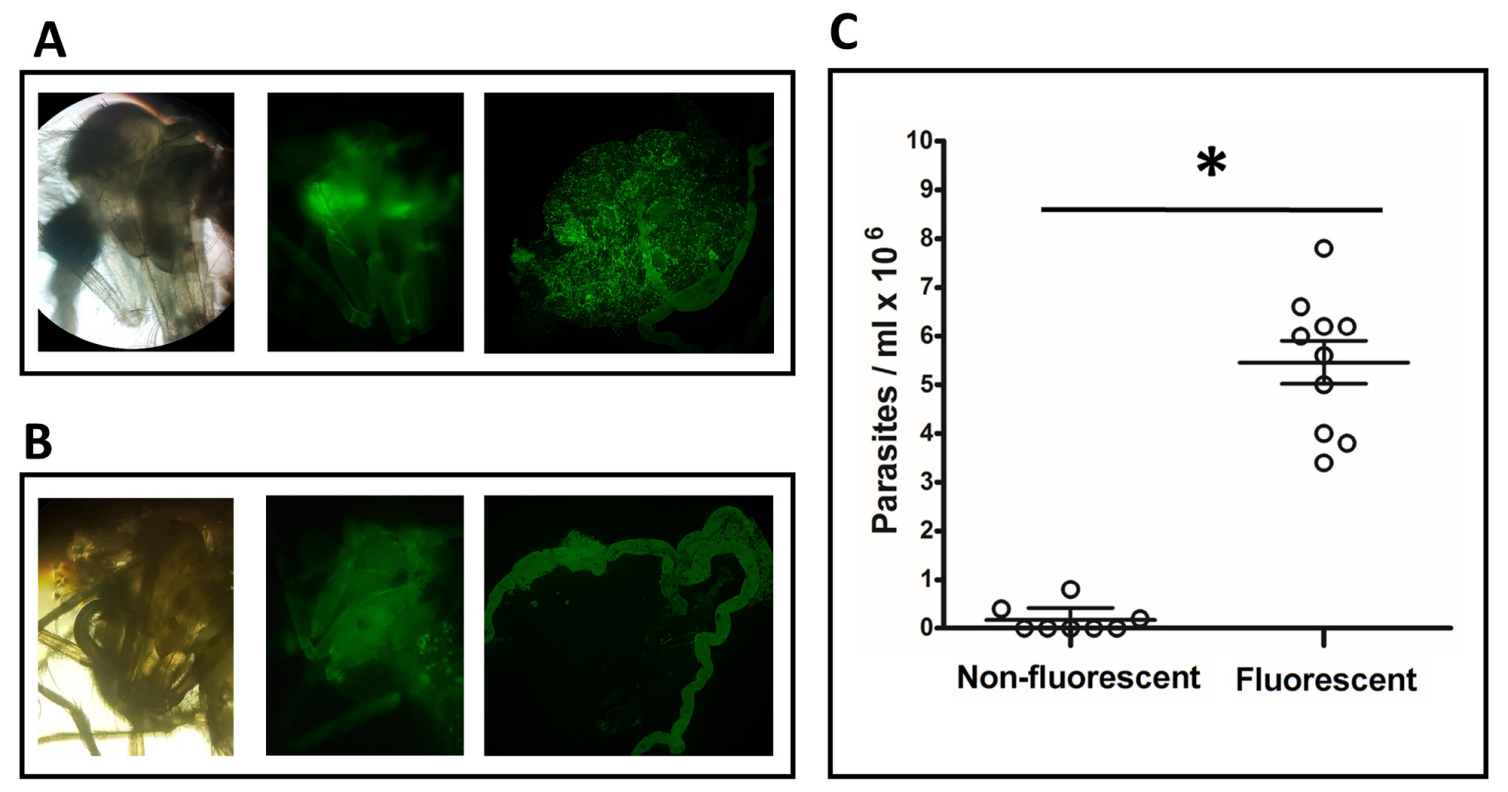
*L. tarentolae*-GFP fluorescence *in vivo*. ( **A**) *L. tarentolae*-GFP Infected individual with localised green fluorescence in the upper thorax: under normal light, under fluorescence, and dissected gut under fluorescence (left to right). ( **B**) *L. tarentolae*-GFP Infected individual without localised green fluorescence in the upper thorax under normal light, under fluorescence, and dissected gut under fluorescence (left to right). ( **C**) Scatter plot shows parasite numbers of *L. tarentolae*-GFP infected flies without localised thorax fluorescence (non-fluorescent) and *L. tarentolae*-GFP infected flies showing localised thorax fluorescence. Each circle represent individual counts, bars are mean ± SEM. Asterisk represents significant differences (unpaired t-test, p<0.005).

Having identified a localised fluorescence in non-dissected *L. tarentolae*-GFP infected flies, 14 flies were sorted under an epifluorescence microscope based on the presence or absence of this feature. Figure 2A shows an infected individual under normal light, under fluorescence, and the gut of the same fly also under fluorescence (left to right). Figure 2B shows an individual that was part of the same *L. tarentolae*-GFP infected group but without localised fluorescence in the thorax region, under normal light, under fluorescence and the gut of that fly also under fluorescence (left to right). Flies that displayed localised fluorescence in the thorax region, and individuals that did not showed this feature, were separated into 2 groups, dissected, and parasites numbers were determined using a Neubauer haemocytometer. Figure 2C shows parasite populations of individuals not showing localised fluorescence vs. individuals not showing localised fluorescence. The number of parasites were significantly higher in flies presenting localised fluorescence compared with those that did not (test p<0.005).

## Discussion

In this work, we successfully infected female *L. longipalpis* artificially with the not-pathogenic to human *Leishmania tarentolae*. We also transfected *L. tarentolae* to express GFP and showed that parasite populations and infection rates were similar between *L. tarentolae* expressing GFP and non-transfected *L. tarentolae*, five days after artificial infection. We also recorded infected sand fly survival with no significant differences between *L. tarentolae*-GFP and *L. tarentolae*-WT. However the key finding of this work was the observation of a distinctive area of fluorescence in non-dissected flies, emitted from inside the thorax, where the midgut is located. Further dissections of sand flies presenting this specific fluorescence were performed and showed comparatively large populations of fluorescent parasites, while flies not showing this feature possessed comparatively very low or no parasite populations.

Our results show that it is possible to use a GFP-expressing non-pathogenic species of *Leishmania* to infect *Lutzomyia longipalpis*. The *L. tarentolae*- *Lu. longipalpis* system provides an additional tool to study vector-parasite interactions using a hazard-free system that can be used to train students, technicians and scientists wishing to study host-parasite interactions. The system may also enable experiments analysing *Leishmania*-resistance and susceptibility in sandflies over the life of the fly. It may also expedite gene silencing experiments on infected flies. Insects where the localised fluorescence was found were cold-anaesthetised and this technique allows recovery of living individuals that can then be kept alive for further experiments. Sand flies show different levels of infection ( Pimenta *et al.*, 1994) and the fluorescence-selection process could allow separation of heavily-infected, low-infected flies and non-infected flies, to rear them as separate groups. This could provide a basis to better understand parasite resistance in sand flies. This technique can also be extended for medically relevant species of Leishmania.

The first experimental infection of sand flies with *L. tarentolae* was performed with *Phlebotomus minutus* fed on infected geckos ( Parrot, 1934). The vectors of this species are *Sergentomyia* spp., and its development in the fly has been described as hypopylarian, this is, a parasite that establishes in the hindgut of the sand fly ( Bates, 2007). Vionette, Ginger & Dillon (unpublished data) showed that *L. tarentolae* could infect *L. longipalpis* and establish a hypopylarian infection. In contrast, our results with the *L. tarentolae*-GFP infection in the fly gut indicated diffuse distribution with no specific binding areas in the gut and did not suggest a hypopylarian type of infection. Regarding our data on *L. tarentolae*-infected flies ( Figure 1C), we found similar results to Rogers & Bates, (2007) who found that *L. mexicana* infections in *Lu. longipalpis* increased insect mortality.

Fluorescence have been extensively used to study several aspects of parasite-vector interactions. Perhaps the largest body of work come from mosquito-malaria and tsetse-trypanosome models. In mosquitoes, the use of fluorescent *Plasmodium* together with several microscopy and imaging techniques has largely contributed understanding parasite dynamics such as ookinete migration and mosquito midgut invasion as well the behaviour of sporozoites during ejection of saliva. These studies have been performed in dissected guts as well as in intact insects or intact body parts ( Frischknecht *et al.*, 2004; Vlachou *et al.*, 2004; Zieler & Dvorak, 2000). Similar experiments performed in tsetse flies have helped to elucidate the establishment of infection by *Trypanosoma brucei*, as well as the dynamics of co-infection with different strains of the parasite in dissected guts ( Gibson & Bailey, 2003). Perhaps some of the most elegant recent work include an extremely detailed modelling of Trypanosome motility, including tsetse tissue topology that resulted in the first 3D modelling if the infection ( Schuster *et al.*, 2017).

Our finding of localised fluorescence present in the upper thorax of *L. tarentolae*-infected flies, and further confirmation of high parasite numbers in these individuals has not been described before for phlebotomines. Given the extensive work carried out in other vector-parasite models, this is a method that should be explored further, building up from some of the many tools available from other models.

Perhaps one of the main limitations of the proposed protocol in this report is the green fluorescence of insect cuticle and Malpighian tubules ( Figure 2A and B), which makes imaging analysis challenging. Qualitative selection of fluorescent and non-fluorescent flies was based on knowledge of the sand fly internal anatomy, as the midgut is pushed forward into the thorax as oocytes increase in size and rests where the green intensity in heavily-infected flies was found. This could be addressed in further experiments evaluating different fluorophores such as DsRed or mCherry to enable a quantitative analysis of fluorescence related to infection intensity.

## Conclusion

Here we report the successful establishment of a sand fly- *Leishmania* model using a species of *Leishmania* non-pathogenic to mammals using GFP-labelled *L. tarentolae*. We also show that infectivity rates on established infections are similar to those achieved using non-labelled parasites. Finally, we show that it was also possible to detect highly-infected and low-infected individuals without the traditional dissection technique, opening a route to enable identification of infected flies for ongoing experimentation.

## Data availability

Figshare: Dataset 1. Non-invasive visualisation and identification of fluorescent Leishmania tarentolae in infected sand flies, https://doi.org/10.6084/m9.figshare.7283546.v1 ( Diaz-Albiter, 2018).

Data are available under the terms of the Creative Commons Zero “No rights reserved” data waiver (CC0 1.0 Public domain dedication).

## Supplementary material

**Supplementary Figure 1. Transfection of *L. tarentolae*.** (A) Diagram of plasmid pGL1132 showing insertion site of GFP. (B) GFP-transfected *L. tarentolae in vitro* (x400).
