# Supplementary figures and images for: Non-invasive visualisation and identification of fluorescent Leishmania tarentolae in infected sand flies
Source: Wellcome Open Res. 2018 Dec 13;3:160. [Version 1] doi: 10.12688/wellcomeopenres.14910.1 (PMC6367660; doi:10.12688/wellcomeopenres.14910.1)

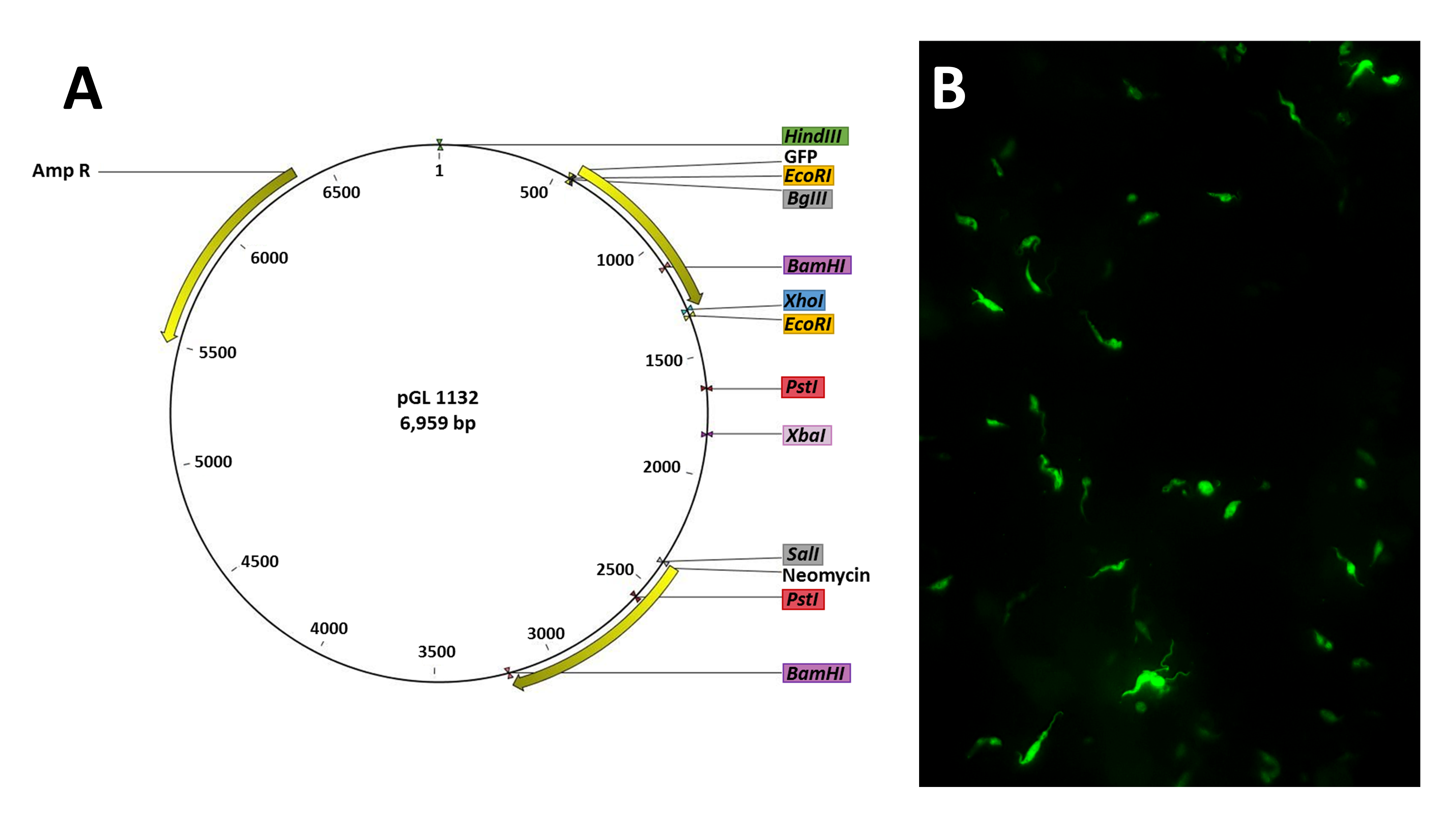

Supplement: Supplementary file 1 [file wellcomeopenres-3-16255-s0000.tgz › d2328713-07dd-4118-9ac3-df3ba5b61c23_Supp_Figure_1.tif]
