# Supplementary material for: Non-invasive visualisation and identification of fluorescent Leishmania tarentolae in infected sand flies
Source: Wellcome Open Res. 2018 Dec 13;3:160. [Version 1] doi: 10.12688/wellcomeopenres.14910.1 (PMC6367660; doi:10.12688/wellcomeopenres.14910.1)
Supplement: Peer review history

# Peer review: Referee response for version 1

**DOI:** 10.21956/wellcomeopenres.16255.r34472

**Published:** 2019-02-07

**Authors:** Antonio Jorge Tempone

**Affiliations:** 1 Laboratório de Biologia Molecular de Parasitos e Vetores-LBMPV, Instituto Oswaldo Cruz - FIOCRUZ, Rio de Janeiro, Brazil

In work titled “Non-invasive visualization and identification of fluorescent *Leishmania tarentolae* in infected sand flies,” the authors developed a system to study interactions between the parasites of *Leishmania* genus and phlebotomine sand flies.  The use of a non-human pathogenic *Leishmania* *tarentolae* favors the students training under better security conditions.  Interestingly, this system allows researchers to quickly and inexpensively identify and isolate highly infected individuals from those with low parasite levels, providing greater sample uniformity.  Besides, the authors identified a decrease in the life span of infected insects, evidencing the harmful nature of *Leishmania* infection.

 Although the authors comment on the use of fluorescent parasites in other parasite-vector interaction models, such as *Plasmodium*-mosquitoes and trypanosomatids-triatomines, there is no citation about the works of Sadlova et al., 2011 ^1^ and Chajbullinova et al., 2012 ^2^. These authors employed fluorescent *Leishmania* in their works. In the first work, the authors verified the existence of genetic material exchange between *L. donovani* during its development in the gut of *Phlebotomus perniciosus* and *Lutzomyia longipalpis*. In the second study, the authors studied the development of *L. turanica *in three different species of phlebotomines. These works should be included in the discussion and references.

I have read this submission. I believe that I have an appropriate level of expertise to confirm that it is of an acceptable scientific standard, however I have significant reservations, as outlined above.

## References

[1] SadlovaJYeoMSeblovaVLewisMDMauricioIVolfPMilesMA: Visualisation of Leishmania donovani fluorescent hybrids during early stage development in the sand fly vector. *PLoS One*.2011;6(5) : 10.1371/journal.pone.0019851 e19851 10.1371/journal.pone.0019851 21637755PMC3103508

[2] ChajbullinovaAVotypkaJSadlovaJKvapilovaKSeblovaVKreisingerJJirkuMSanjobaCGantuyaSMatsumotoYVolfP: The development of Leishmania turanica in sand flies and competition with L. major. *Parasit Vectors*.2012;5: 10.1186/1756-3305-5-219 219 10.1186/1756-3305-5-219 23031344PMC3484061

# Peer review: Referee response for version 1

**DOI:** 10.21956/wellcomeopenres.16255.r34465

**Published:** 2019-01-10

**Authors:** Yusuf Özbel

**Affiliations:** 1 Department of Parasitology, Faculty of Medicine,, Ege University, İzmir, Turkey

In this study, the researchers developed a system for investigating the interactions between sand fly species and Leishmania parasites.

 In my opinion, it is very useful, especially for training, and if it is extended to human pathogenic Leishmania species, we can better understand the events happening in the vector sand fly species. This information can give us the explanations why some Leishmania species are specific to some sand fly species and vice versa.

 The manuscript is very well written and I do not have any corrections.

 It can be accepted as it is. 

I have read this submission. I believe that I have an appropriate level of expertise to confirm that it is of an acceptable scientific standard.

# Peer review: Referee response for version 1

**DOI:** 10.21956/wellcomeopenres.16255.r34466

**Published:** 2019-01-09

**Authors:** Jesus G Valenzuela

**Affiliations:** 1 Laboratory of Malaria and Vector Research, National Institute of Allergy and Infectious Diseases, National Institutes of Health (NIH), Rockville, MD, USA

The work by Diaz-Albiter et al describes the development of a safe system to study Leishmania-sand fly interactions that will not need a special containment facility. The group developed a green fluorescent *Leishmania tarentolae*parasite, infected sand flies with these parasites and observed the parasites inside of the gut of the sand fly. They also compared the survival in sand flies of the green fluorescent parasites and the wild type parasites. 

 The work is relevant particularly because this system will allow vector-parasites studies in a system that does not require stringent biosafety level of containment. There are some questions the authors need to address and some changes to do in the manuscript:

  

1. In the abstract GFP should be in parenthesis (GFP).
2. Introduction: “argentypes” change to “argentipes”.
3. Introduction: “well-establish” change to “well-established”.
4. Did the authors follow the infection after 5 days post infection?
5. What parasites stages do the authors observe in the sand flies at 5 days post infection?
6. By not extending the days of infection the authors are missing a very important time point, the emergence of metacyclics. 
7. This system can be used also to test transmission blocking vaccines.
8. Methods: change “lab” to “laboratory”.
9. The authors use sometimes “sandfly” and other times “sand fly” throughout the manuscript, this needs to be consistent for whatever the authors decide to use.
10. Figure 1A.  The Y axis numbers do not make much sense.  Can the authors write how many parasites were found per insect or per midgut? If the numbers are in the millions please check your calculations.
11. Same for Figure 2C. Can the authors write how many parasites were found per insect or per midgut?  If the numbers are in the millions please check your calculations.
12. Figure 1B. I would flip the graph. Show the infected in the bottom and the uninfected in the top. This will match the label of the Y axis. Otherwise it is very confusing.
13. Under “Assessment of L. tarentolae-GFP fluorescence in vivo”, change:  “did not showed” to “did not show”
14. Discussion.  The authors mentioned “This technique can also be extended for medically relevant species of Leishmania”.  This was actually already shown by Sacks et al using L *. major*fluorescent parasites and by Guevara P et al. (2001) ^1^. I think the authors should provide the references of Leishmania fluorescent parasites being previously used in sand flies. What the presented work emphasizes is actually having a safe system to study vector-parasite interactions using fluorescent parasites. The use of Leishmania fluorescent parasite is not novel and it should be mentioned more in this paper, particularly in the paragraph where the authors describe the *Plasmodium *and *Trypanosome *work *. *

I have read this submission. I believe that I have an appropriate level of expertise to confirm that it is of an acceptable scientific standard, however I have significant reservations, as outlined above.

## References

[1] GuevaraPPinto-SantíniDRojasACrisanteGAñezNRamirezJL: Green fluorescent protein-tagged Leishmania in phlebotomine sand flies. *J Med Entomol*.2001;38(1) :39-43 1126868910.1603/0022-2585-38.1.39
